# Adaptation of Clinical Prediction Models for Application in Local Settings

**DOI:** 10.1177/0272989X12439755

**Published:** 2012-05

**Authors:** Teus H. Kappen, Yvonne Vergouwe, Wilton A. van Klei, Leo van Wolfswinkel, Cor J. Kalkman, Karel G. M. Moons

**Affiliations:** Division of Anesthesiology, Intensive Care and Emergency Medicine (THK, WAVK, LVW, CJK, KGMM), University Medical Center Utrecht, Utrecht, the Netherlands; Julius Center for Health Sciences and Primary Care (YV, KGMM), University Medical Center Utrecht, Utrecht, the Netherlands

**Keywords:** clinical prediction rules, methodology, decision rules, provider decision making, statistical methods

## Abstract

**Background.** When planning to use a validated prediction model in new patients, adequate performance is not guaranteed. For example, changes in clinical practice over time or a different case mix than the original validation population may result in inaccurate risk predictions. **Objective.** To demonstrate how clinical information can direct updating a prediction model and development of a strategy for handling missing predictor values in clinical practice. **Methods.** A previously derived and validated prediction model for postoperative nausea and vomiting was updated using a data set of 1847 patients. The update consisted of 1) changing the definition of an existing predictor, 2) reestimating the regression coefficient of a predictor, and 3) adding a new predictor to the model. The updated model was then validated in a new series of 3822 patients. Furthermore, several imputation models were considered to handle real-time missing values, so that possible missing predictor values could be anticipated during actual model use. **Results.** Differences in clinical practice between our local population and the original derivation population guided the update strategy of the prediction model. The predictive accuracy of the updated model was better (c statistic, 0.68; calibration slope, 1.0) than the original model (c statistic, 0.62; calibration slope, 0.57). Inclusion of logistical variables in the imputation models, besides observed patient characteristics, contributed to a strategy to deal with missing predictor values at the time of risk calculation. **Conclusions.** Extensive knowledge of local, clinical processes provides crucial information to guide the process of adapting a prediction model to new clinical practices.

Prediction models allow clinicians to estimate a patient’s prognosis or a patient’s probability of having a particular diagnosis.^1,2^ Clinicians are increasingly using prediction models in clinical practice to guide their decision making. Prior to its use, clinicians should decide whether the model will provide accurate predictions for their patients.^1,3–5^

At least two possible scenarios should be considered. First, the local population may differ from the derivation population or the model is no longer up to date with current scientific knowledge. Using the original prediction model without an adaptation to local circumstances may render inaccurate predictions in the new patients. Second, predictor values may be missing when the model is actually being used to calculate risks (real-time missings). For example, the test or device to assess a predictor’s value is not available. Simply leaving the predictor out of the model will lead to inaccurate predictions.

In recent methodological literature, strategies have been developed to update an existing prediction model to overcome differences between the derivation and local populations.^6,7^ Update techniques aim to improve the performance of the original model in new patients by using additional patient information of the local population. The information that is already available in the original model is retained rather than discarded.^8^ Knowledge on differences in case mix or changes in clinical practice over time may allow for specific adjustments to a subset of predictors, in addition to an overall adjustment to the original model.

Furthermore, strategies have been reported to handle real-time missing predictor data when a prediction model is used in new patients.^9^ Recently suggested methods to handle real-time missing predictor data are mostly based on statistical techniques. Clinicians—or other health care workers—may be aware of specific reasons why predictor data are missing. For example, logistics can cause missing values when a predictor’s value is only available in a paper-based format, while the predicted risk is automatically being calculated by an electronic decision support system. Consequently, this local, external information may be combined with statistical techniques to provide accurate estimates of missing predictor values in the local population.

This case study demonstrates how we used insight in recent developments and local circumstances to optimize a model for predicting postoperative nausea and vomiting (PONV) before using the model in our clinical practice. Specific antiemetic drugs can be administered during anesthesia to prevent PONV, a practice known as “PONV prophylaxis.”^10^ For high-risk patients, several antiemetic drugs need to be combined to effectively prevent PONV. A prediction model might enable clinicians to apply a risk-tailored approach for PONV prophylaxis and prevent unnecessary costs and possible side effects, in contrast to administering multiple drugs to all patients.^11,12^

## Methods

### The Original PONV Prediction Model

Previously, a prediction model for PONV occurrence within 24 hours of surgery was derived and internally validated.^13^ Data were used of 1389 inpatients who were scheduled for elective surgery between 1997 and 1999 in the Academic Medical Center Amsterdam, the Netherlands (hereafter referred to as derivation data set). All patients had participated in a randomized trial on differences in PONV incidence after either intravenous or inhalational general anesthesia.^14^ The model is shown in Table 1, left column. Initially, 7 categories were used to describe data on type of surgery: superficial, laparoscopic, upper abdominal, lower abdominal, strabismus, middle ear, and other. As the risk of PONV was significantly higher for lower abdominal surgery and middle ear surgery compared with all other categories in the derivation data set, both were combined into high-risk surgery v. all other types of surgery.

**Table 1:** Regression Coefficients for Predictors and Intercept of the Original Prediction Model, the Updated Prediction Model, and the New Prediction Model.

Predictor	Original Model	Updated Model	Newly Developed Model
Age, y	−0.022	−0.017	−0.010
Female gender	0.46	0.36	0.63
Current smoking	−0.63	−0.50	−0.068
History of PONV or motion sickness	0.76	0.60	0.85
Lower abdominal or middle ear surgery	0.61	—	—
Abdominal or middle ear surgery^a^	—	0.48	0.63
Isoflurane and/or nitrous oxide anesthesia^b^	0.72	—	—
Inhalational anesthesia^c^	—	0.35	0.33
Outpatient surgery	—	−1.16	−1.14
Intercept	0.15	0.12	−0.65

PONV, postoperative nausea and vomiting.

a. In the updated model and newly developed model, this predictor replaced “lower abdominal or middle ear surgery” from the original model. In the updated model and the newly developed model, it included lower abdominal, upper abdominal, and laparoscopic surgery in addition to middle ear surgery.

b. As compared with intravenous anesthesia using propofol.

c. As compared with intravenous anesthesia using propofol. In the updated model and newly developed model, this predictor replaced “isoflurane and/or nitrous oxide anesthesia” from the original model.

d. Predictor not included in the original model.

### Updating the PONV Prediction Model

#### Clinical considerations

The decision to update the original model was based on 3 clinical considerations. First, we compared the predictors in the model with the latest reported evidence on important PONV predictors. Second, we considered possible changes in anesthesia practice since the model derivation. Third, we considered possible differences in case mix between the derivation data set and our local patients.

According to recent literature, upper abdominal and laparoscopic surgery are procedures that have a risk of PONV similar to lower abdominal surgery, as all 3 types of procedures are forms of abdominal surgery.^15,16^ Upper abdominal and laparoscopic surgery were not included in the original model because of small numbers in the derivation data set. Hence, we decided to change the definition of the predictor high-risk surgery to “middle ear surgery or any type of abdominal surgery”.

The derivation data set included patients who had surgery in the middle to late 1990s. At that time, nitrous oxide was commonly used as an additive agent to ether derivatives for inhalational anesthesia. Both ether derivatives and nitrous oxide increase the risk of PONV. Currently, nitrous oxide is much less used in the Netherlands, in response to the discovery of several adverse effects, including PONV.^17^ The main ether derivative of that time, isoflurane, is increasingly being replaced by new, shorter acting inhalational agents such as sevoflurane and desflurane, which are considered to cause less PONV.^18^ As predictive effects of inhalational anesthesia could be different at the present time, we decided to include the changes in anesthesia practice in the definition of the predictor inhalational anesthesia and its regression coefficient in the model.

The original prediction model was derived on surgical inpatients only. In general, PONV is less common in outpatient procedures than in inpatient procedures. Consequently, the original PONV model was likely to overestimate the risk of PONV for outpatients. The lower incidence in outpatients is most likely the result of a lower case complexity. However, case complexity is a result of several characteristics, which individually are not necessarily predictors of PONV. Outpatient surgery may therefore serve as a proxy variable in which the predictive effects of several of these case characteristics are combined. Since outpatient surgery is currently routine practice in many hospitals, including ours, we decided to extend the model with an additional predictor for outpatient surgery.

#### Update data set

Using a prospective cohort design, we consecutively included 1847 elective surgical patients who were operated on from June to December 2004 under general anesthesia at the University Medical Center Utrecht, the Netherlands (referred to as update data set). The study included adult inpatients (*n* = 1203) and outpatients (*n* = 644). All predictor variables of the original model and the outcome variable PONV within 24 hours were recorded similar to the data collection of the derivation data set.

#### Update methods

The original prediction model was updated with logistic regression analysis. For every patient in the update data set, the linear predictor (*lp*_0_) was calculated based on the 6 regression coefficients (β_*i*_) of the original model and the corresponding predictor values (*x*_i_) of that case (*lp*_0_=α+ β_1_*x*_1_*+* β_2_x_2_*+. . .+* β_6_x_6_*)* (see Table 1 for predictor variables). According to clinical considerations as described above, the model was updated using recently proposed methods.^6,7^ According to recent literature, upper abdominal and laparoscopic surgery would have predictive effects of PONV similar to lower abdominal surgery. Consequently, we assumed that the change in definition of high-risk surgery would have the same regression coefficient as compared with the old definition. In contrast, the change in definition of inhalational anesthesia was expected to lower its regression coefficient. Therefore, the model was revised for inhalational anesthesia (*x*_inhalational_). To include outpatient surgery, the model was extended with an additional predictor (*x*_outpat_). The intercept (α) and slope (β_overall_) were estimated to incorporate the overall effect of the new data. This resulted in the updated model with the following linear predictor (*lp*_1_):

$$lp_{1}=\alpha +\beta_{\text{overall}}lp_{0}+\gamma_{\text{inhalational}}x_{\text{inhalational}}+\beta_{\text{outpat}}x_{\text{outpat}}.$$

In formula (1), γ_inhalational_ represents the deviation from the recalibrated regression coefficient for inhalational anesthesia, and β_outpat_ represents the regression coefficient for the extra predictor outpatient surgery (yes/no). All regression coefficients of the original model were multiplied by β_overall_ to result in the updated (recalibrated) regression coefficient. In addition, γ_inhalational_ was added to the recalibrated regression coefficient of inhalational anesthesia to result in its final regression coefficient in the updated model.

For comparison purposes, a new model was developed from the update data set using the same predictor variables as the updated model. However, the regression coefficients for the newly developed model were estimated directly from the update data set and did not include any information from the original data set. Consequently, the regression coefficients of the newly developed model represented the “closest fit” to the update data set. The new model was used to evaluate the success of adapting the updated model to the local setting.

Multiple imputation was used to impute missing data within the update data set (aregImpute [Vanderbilt University, Nashville, TN] from the Hmisc library of R [Free Software Foundation, Boston, MA] and S-plus [TIBCO Software Inc. Palo Alto, CA] software). Regression coefficients of formula (1) and regression coefficients of the newly developed model were estimated in 10 completed data sets and averaged.^19–21^

#### Validation of updated model

In the next phase, the updated model and the newly developed model were externally validated in a third cohort of 3822 elective surgical in- and outpatients, prospectively collected between March 2006 and February 2007 at the University Medical Center Utrecht, the Netherlands (referred to as external validation data set). Following multiple imputation of missing data, receiver operating characteristic (ROC) curves were plotted to compare the predictive performance of all models within the external validation data set. Discrimination was further assessed with the c statistic.^22–24^ Calibration within the external validation data set was assessed with calibration intercept and slope.

### Handling of Real-Time Missing Predictors

When either the updated model or the newly developed model will be used in our local population, various predictor values are likely to be missing on occasion. Similar to dealing with missing values when analyzing the data of a conducted study, missing predictor values should also be dealt with during model application (i.e., in real time). We developed an imputation strategy that can be used to estimate missing predictor values when applying the prediction model in practice. The strategy contains various imputation models.

At the start of a procedure (i.e., the moment of risk calculation), the predictors age, gender, inhalational anesthesia, and outpatient surgery are always available from the electronic patient record and will never be missing. We derived imputation models for the predictors high-risk surgery, smoking status, and history of PONV or motion sickness using data from the update data set. Although the planned surgical procedure is always known at the start of the procedure, whether it is a high-risk surgery or not requires some additional recoding to use it as a predictor in an electronic decision support system. When the information has not yet been recoded at the moment of risk calculation, causing “high-risk surgery” to be missing, the observed values of other predictors alone may not be informative enough to impute missing values on high-risk surgery. Two process variables, which indicate the nature of the surgical procedure and are always electronically available at the start of the procedure, were therefore added: surgical specialty of the treating clinician and operating room location. Surgical specialty provides valuable information about whether high-risk surgery is being performed. For example, gynecologists often perform abdominal surgical procedures, whereas ophthalmologists never perform abdominal or middle ear procedures. Consequently, a surgical specialty is considered a risk specialty if either abdominal or middle ear surgery is included in the regular scope of procedures of that specialty. When surgical specialty is missing as well, the probability of a surgical specialty performing high-risk surgery will be estimated from the particular operating room. Surgical specialties are mostly restricted to one or two specific operating rooms. More than 50,000 electronic patient records on procedures in the 4 years preceding the study were used to determine if an operation room should be considered to accommodate surgical specialties that perform high-risk surgery. Subsequently, either the surgical specialty or the probability of a surgical specialty that performs high-risk surgery was included into the imputation model for high PONV risk surgery, in addition to the available predictors. The ability to predict surgical specialty from the location of the operating room was assessed from the external validation data set using the c statistic, as well as the calibration intercept and slope.

The imputation strategy as a whole was validated in cases with completed outcome data of the external validation data set. The 3 predictors—high-risk surgery, smoking status, and history of PONV or motion sickness—were imputed. The PONV risk was calculated with the imputed values for all 3 predictors. The performance of the updated model using our imputation strategy was compared with the performance of the updated model using 2 alternative strategies to dealing with real-time missing predictors: overall mean imputation (impute the data set incidence for each predictor value) and ignoring the missing predictor (set predictor value to zero). Reclassification of the imputation strategy in comparison to overall mean imputation was expressed as a category-free net reclassification improvement (NRI [>0]) with bootstrapped 95% confidence intervals (boot library of R and S-plus software).^25,26^

## Results

### Update of the Prediction Model

The calibration slope (β_overall_) of the original model was 0.79, with an intercept adjustment (α) of −0.03 (see formula (1)). The regression coefficient correction for inhalational anesthesia (γ_inhalational_) was −0.22. As a consequence, the new regression coefficient for inhalational anesthesia was 0.35 (0.79*0.72-0.22, Table 1, right column). The additional reduction of the predictive effect was expected since newer inhalational agents and the less frequent use of nitrous oxide had reduced the incidence of PONV. Outpatient surgery was less frequently related to PONV than inpatient surgery, as expressed by a regression coefficient of −1.16 (β_outpat_).

The distributions of the patient characteristics in the update data set and the external validation data set were similar for most variables (Table 2). Patients from the update data set were slightly younger (mean: 47 v. 50 years), received more often inhalational anesthesia (61% v. 52%), and underwent outpatient surgery more often (35% v. 24%) compared with patients from the external validation data set. Predictor values in the update data set were missing in 23% of the cases for history of PONV, in 18% for motion sickness, and in 1% for current smoking. For the external validation data set, missings occurred in 30% of the cases for history of PONV, in 29% for motion sickness, in 7% for type of surgery, and in 4% for current smoking. Patients from the update data set suffered less frequently from PONV (35% v. 47%), which is probably the result of the higher number of outpatients in the update data set. Outcomes were missing in 6% of the cases in the update data set and in 25% of the cases in the external validation data set. The larger percentage of missing outcomes in the external validation data set was caused by a technical problem in (the logistics of) the data collection.

**Table 2:** Patient and Procedural Characteristics of the Update Data Set and the External Validation Data Set

	Update Data Set (*n* = 1847)	External Validation Data Set (*n* = 3822)
Age, y, mean (SD)	47 (16)	50 (17)
Female gender	1023 (55)	2059 (54)
Current smoking	595 (32)	1145 (31)
History of PONV	452 (25)	737 (28)
Motion sickness	182 (10)	234 (9)
High-risk surgery	292 (16)	546 (15)
Inhalational anesthesia	1126 (61)	1971 (52)
Outpatient surgery	644 (35)	928 (24)
PONV occurrence within 24 hours	653 (35)	1340 (47)

Values are presented as No. (%), unless stated otherwise. PONV, postoperative nausea and vomiting.

**Figure 1.**
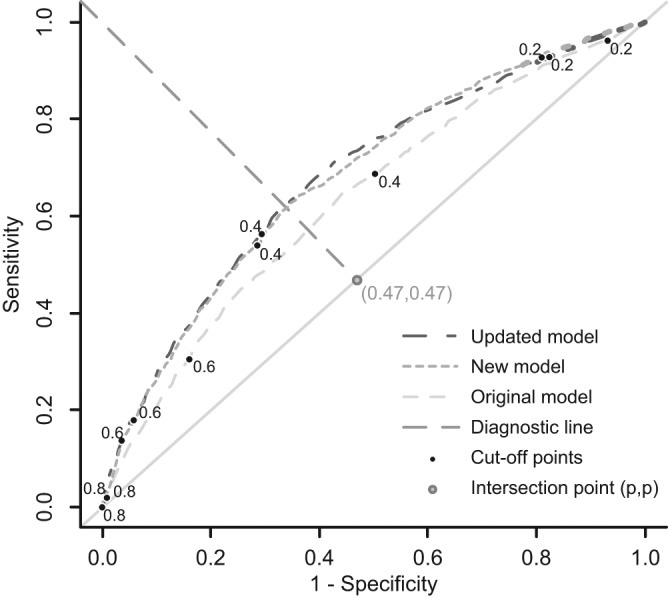
Receiver operating characteristic curves of all 3 prediction models in the external validation data set. Numbers near cutoff points indicate the predicted risks related to the cutoff points. Point (p,p) is the point with the proportion of patients who experienced postoperative nausea and vomiting (*P* = 0.47). The point (p,p) and point (0,1) were used to construct the diagnostic line.

In the external validation data set, discrimination was very similar for the updated model and the newly developed model and lower for the original model (Figure 1). Application of the updated model in the patients of the external validation data set resulted in a c statistic of 0.68 (95% confidence interval [CI], 0.66–0.70) and a calibration slope of 1.00 (95% CI, 0.89–1.10) with a calibration intercept of 0.34. When applying the newly developed model in the external validation data set, the model had a c statistic of 0.68 (95% CI, 0.67–0.70) and a calibration slope of 0.89 (95% CI, 0.80–0.99) with a calibration intercept of 0.29. For comparison purposes, the c statistic of the original model in the external validation set was 0.62 (95% CI, 0.60–0.64), and the calibration slope was 0.57 (95% CI, 0.48–0.66).

### Handling of Real-Time Missing Predictors

**Figure 2.**
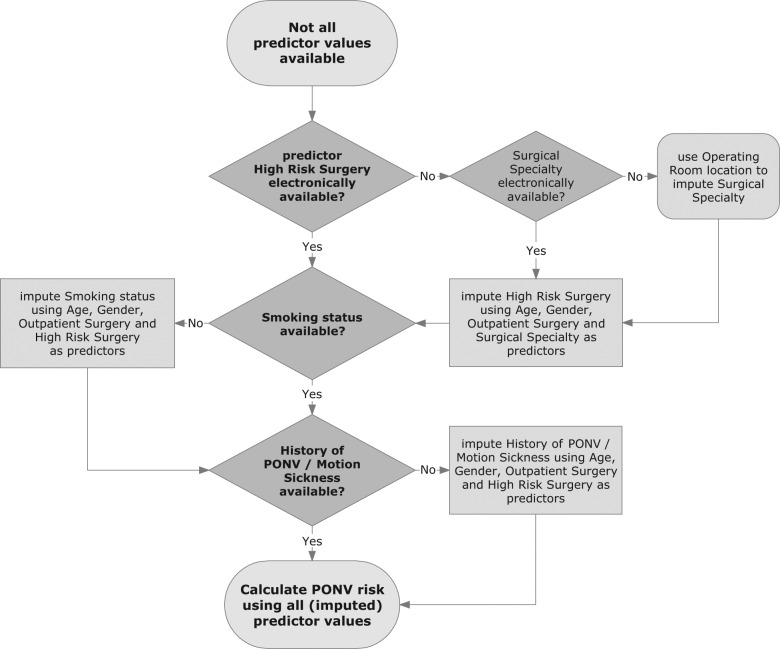
Flowchart of the imputation strategy for real-time missing predictors. PONV, postoperative nausea and vomiting.

The imputation strategy incorporated 3 imputation models: 1 model for high-risk surgery, 1 for smoking status, and 1 for history of PONV or motion sickness (Figure 2). First, high-risk surgery was imputed based on observed predictors and additional process variables. As a consequence, the additional information was indirectly incorporated in the imputation of missing values on smoking status and history of PONV or motion sickness. The value of high-risk surgery was imputed with a logistic regression model that included age, gender, outpatient surgery, and surgical specialty as variables. When surgical specialty was unavailable, it was estimated from the location of the particular operating room, with a c statistic of 0.96 (95% CI, 0.95–0.96), a calibration slope of 1.03 (95% CI, 0.97–1.10), and a calibration intercept of 0.68. Subsequently, missings on smoking status and history of PONV or motion sickness were estimated with imputation models that included the variable high-risk surgery, in addition to age, gender, and outpatient surgery. The predictor inhalational anesthesia was not included in any of the imputation models, as a physician generally decides on the type of anesthesia after calculating the PONV risk. Regression coefficients of the imputation models are not presented because they reflect the local situation of our hospital. Using the imputation strategy, the updated model showed a c statistic of 0.67 (95% CI, 0.65–0.69) and a calibration slope of 1.00 (95% CI, 0.86–1.13) with a calibration intercept of 0.54. For the other 2 strategies, the c statistic (0.65; 95% CI, 0.63–0.67) and calibration slope (0.98; 95% CI, 0.84–1.13) were slightly lower, with a small difference in calibration intercept (0.54 for overall mean imputation v. 0.65 for ignoring predictor). In comparison to overall mean imputation, the total NRI (>0) for the imputation strategy was 44% (95% CI, 37%–51%), with 33% within patients with PONV and 11% within patients without PONV. With the imputation strategy ready, the updated PONV prediction rule was further optimized for future use in our hospital.

## Discussion

This study demonstrates the great value of information on the local patient population and local clinical practice, at the time that a prediction model is going to be used in that practice. In addition, potential users of clinical prediction models need to consider not only possible differences between the derivation population and the local population in terms of case mix but also newly published evidence that is relevant to their patient population and changes in clinical practice over time. We used local information and scientific knowledge to adapt a prediction model to our local setting (i.e., extending the definition of one of the predictors, adjusting the predictive effects, and adding a new predictor). The updated prediction model showed much better predictive performance in an external validation data set as compared with the original model. A newly developed model (i.e., without incorporating information of the original model) showed discrimination in the external validation data set similar to the updated model, but calibration was not as good as the updated model. Furthermore, information on local logistics was used for the development of a real-time imputation strategy when predictor values are missing in clinical practice.

When we considered using a prediction model for PONV in our clinical practice, we encountered 3 situations that required adjustment of the original prediction model. First, we extended the definition of one predictor because we expected that the scope of the predictor would be different in our local population compared with the derivation population. We left the predictive effect of the predictor unchanged. In our example, 2 types of abdominal procedures were known from the literature to be important risk procedures, with PONV risks similar to procedures of the original index category of the predictor high-risk surgery. Due to an absent association in the derivation data set, the 2 procedures had not been included in the original index category. As the literature considered the additional procedures to be high-risk procedures, we extended the definition of high-risk surgery without reestimating its regression coefficient.

Second, the effect of a predictor was expected to be different for our local population than for the derivation population.^5–8^ New inhalational anesthetics had become available since the derivation of the original prediction model. Consequently, the original estimate of the predictor effect would no longer be appropriate, resulting in incorrect risk estimates in our patients. We updated the original regression coefficient of the predictor inhalational anesthesia to include the new inhalational agents into the definition of the predictor.

Third, as our local population included an additional subset of patients at a lower risk of PONV (outpatients), a new predictor was added to the prediction model. We assumed that the new predictor did not disturb proportionality between regression coefficients of the original predictors. The additional predictor enabled us to use the prediction model not only for our inpatient procedures but also for our outpatient procedures.

Real-time missings of predictor data are likely to be an issue during actual use of any prediction model in daily practice.^9^ Relying solely on observed predictor values to estimate the missing predictor values will probably not render the best predictions for those patients. Clinicians–or other health care workers—may be aware of variables that might serve as proxy for the missing predictor. In our study, logistics were expected to cause missing values for the predictor high-risk surgery. Our strategy for real-time imputation of missing predictors prevented a drop of 0.02 in the c statistic (0.67 v. 0.65) as compared with overall mean imputation and ignoring the predictor. This 0.02 drop in the c statistic may be considered a relevant decrease in the accuracy of predicted risks of individual patients since the c statistic is relatively insensitive. The high net reclassification improvement confirms this result.^25,26^ Hence, we believe that developing the imputation strategy was worth the effort, as it is likely that such a strategy improves PONV risk prediction by using “the best guess” for that patient’s value using proxy surgery variables.^9^

The goal of updating the PONV prediction model was to adapt the original model to the current clinical practice of the anesthesia department in our hospital. We successfully adapted the model to our clinical practice, as confirmed by the predictive performance of the updated model in the external validation sample. Discrimination of the updated model was similar to previous validation studies of (other) PONV prediction models (c statistic of 0.68) and was better than discrimination of the original model (c statistic of 0.62).^27–29^ Moreover, the calibration slope of the updated model was 1.00, whereas it was only 0.57 for the original model. The importance of a better calibrated model is reflected in the use of the model in clinical practice. Using a patient’s individual PONV risk as predicted by the prediction model, a clinician may decide to administer specific antiemetic drugs during the procedure that may reduce the risk of PONV. Each class of these antiemetic drugs reduces the risk by a relative 25%, with an additive effect for each additional class administered.^10^ It is important for clinicians to accurately know their patients’ individual PONV risks to weigh the benefit of administering 1 or more antiemetic drugs against possible side effects of the drugs. Unfortunately, the updated prediction model systematically underestimated the actual PONV risk in the external validation data set (reflected by a calibration intercept of 0.34). An important reason for the systematic miscalibration is the difference in PONV incidence between the external validation data set and the update data set (log([0.47/0.53]/[0.35/0.65]) = 0.50) on the log-odds scale). The calibration intercept of 0.34 is less than the 0.50 of the crude incidence difference on the log-odds scale. As the calibration intercept did not fully cover the crude incidence difference, the difference in PONV incidence was partly resolved by the predictors in the model (0.50 – 0.34 = 0.16 on the log-odds scale). Since underestimation can easily be adjusted for by adapting the model intercept,^30,31^ the updated model is preferred over the original model.

Although it might seem intuitive that a prediction model, specifically developed in a local setting, will perform better in the local population than an update or adjustment of an existing model to that local situation, the updated model might actually be preferred over a newly developed model because of improved generalizability.^5–7^ This is also reflected by our results. In new patients of the same institute (the external validation data set), the updated model actually performed better than the newly developed model, notably in terms of calibration. A second indication of improved generalizability of the updated model over the newly developed model can be observed from the absence of a predictive effect in the new data set for “current smoking”. As current smoking is a consistent predictor of PONV throughout literature,^12^ the absence of a predictive effect of current smoking seems implausible.

This case study is an example of how a prediction model can be adapted to local circumstances. One limitation is that the required adaptations will not be the same for all prediction models and clinical settings. For example, differences in case mix may specifically occur between derivation and local populations, whereas all prediction models will be subject to some degree of change in clinical practice over time. Second, this study does not provide an exhaustive summary of all possible techniques to enhance updating of a prediction model or to handle missing predictor data when applying a prediction model in real time. Alternative methods to handle real-time missing predictor data have been summarized recently.^9^ Third, we updated the PONV prediction model to derive accurate predictions for our own patients. We cannot conclude from this study whether use of the updated model in clinical practice will be successful. A formal impact study should be performed to evaluate the effects of the prediction model on clinical practice and patient outcome.^5,32,33^ Finally, individual patient data were required to update the prediction model and to develop the imputation strategy. Owing to a previous study within our hospital, data were already available at the time we planned to use the model. Still, the update data set was too small to develop imputation models for high-risk surgery using the proxy variables and required an additional data set to estimate surgical specialty.

To conclude, the intention to use a prediction model in new patients should trigger researchers and clinicians to decide whether they indeed expect an accurate performance of the prediction model in their local population. In our example, we expected problems with generalization of the original prediction model to our patients, despite previous validations of the model. An update of the model proved most valuable to improve its performance in our current population, and a strategy to handle missing predictor data enabled us to actually use the model in clinical practice, even when a predictor value would be missing. As these problems with generalization and application of clinical prediction models are likely to be common, it is conceivable that any prediction model will need to be optimized before it is actually used in a new setting. When optimization is indeed required, a sufficiently large data set from the local population should be readily available or will have to be collected. Clinical insights into the lack of generalization will have to be combined with statistical methods to optimize a prediction model’s performance in future patients.
